# Pharmacological Inhibition of the Nucleus Accumbens Increases Dyadic Social Interaction in Macaques

**DOI:** 10.1523/ENEURO.0085-24.2024

**Published:** 2024-04-16

**Authors:** Hannah F. Waguespack, Jessica T. Jacobs, Janis Park, Carolina Campos-Rodriguez, Rafael S. Maior, Patrick A. Forcelli, Ludise Malkova

**Affiliations:** ^1^ Interdisciplinary Program in Neuroscience, Georgetown University, Washington, DC 20007; ^2^ Department of Pharmacology & Physiology, Georgetown University, Washington, DC 20007; ^3^Laboratory of Neurosciences and Behavior, Department of Physiological Sciences, Institute of Biology, University of Brasilia, Brasilia 70.910-900, Brazil; ^4^Department of Neuroscience, Georgetown University, Washington, DC 20007

**Keywords:** dominance, dopamine, GABA, muscimol, quinpirole

## Abstract

The nucleus accumbens (NAc) is a central component of the brain circuitry that mediates motivated behavior, including reward processing. Since the rewarding properties of social stimuli have a vital role in guiding behavior (both in humans and nonhuman animals), the NAc is likely to contribute to the brain circuitry controlling social behavior. In rodents, prior studies have found that focal pharmacological inhibition of NAc and/or elevation of dopamine in NAc increases social interactions. However, the role of the NAc in social behavior in nonhuman primates remains unknown. We measured the social behavior of eight dyads of male macaques following (1) pharmacological inhibition of the NAc using the GABA_A_ agonist muscimol and (2) focal application of quinpirole, an agonist at the D2 family of dopamine receptors. Transient inhibition of the NAc with muscimol increased social behavior when drug was infused in submissive, but not dominant partners of the dyad. Focal application of quinpirole was without effect on social behavior when infused into the NAc of either dominant or submissive subjects. Our data demonstrate that the NAc contributes to social interactions in nonhuman primates.

## Significance Statement

A range of neuropsychiatric disorders present with altered reward processing and social behavior. The nucleus accumbens (NAc) is a critical brain region for reward processing and motivated behavior. While prior studies in rodents have suggested a role for the NAc in social behaviors, no studies have examined this role in the primate brain. Here, we bridge this gap and demonstrate that focal modulation of activity within the primate NAc alters social behavior and that this effect is modulated by the social status of the infused animal.

## Introduction

The nucleus accumbens (NAc), sometimes referred to as the ventral striatum, is a central integrator of cortical and limbic inputs and is thought to play a critical role in goal-directed behaviors (Sesack and Grace, 2010; Salgado and Kaplitt, 2015). The NAc receives extensive cortical and thalamic inputs (Powell and Leman, 1976), as well as dopaminergic input from the ventral tegmental area (VTA; Watabe-Uchida et al., 2012).

Given the central role the NAc plays in reward processing (Stratford and Kelley, 1997; Peciña and Berridge, 2005; Day and Carelli, 2007; Schlaepfer et al., 2008; Stuber et al., 2011; Trezza et al., 2012; van der Plasse et al., 2012; Van Zessen et al., 2012; Dölen et al., 2013), and given the importance of reward processing to social behavior (Insel, 2003), it follows that the NAc is of growing interest in the context of social behavior. In rodent models, manipulation of the NAc impacts a range of social domains. For example, lesions of the NAc abolish socially transmitted food preference in rats (Noguer-Calabús et al., 2022), and transient pharmacological inhibition of the NAc by GABA agonists increases social play in rats (Van Kerkhof et al., 2013) and decreases submissive behavior following social defeat, that is, “conditioned defeat,” in Syrian hamsters (Luckett et al., 2012). Consistent with this, chemogenetic activation of NAc neurons modulates social dominance in mice (Shan et al., 2022).

The only study to date examining the NAc in the context of social behavior in primates was in titi monkeys, a species of monogamous primates. PET imaging indicated that pair-bonding is associated with decreased activity in the NAc, with lone males displaying heightened glucose uptake as compared with pair-bonded males (Bales et al., 2007).

Within the NAc, dopaminergic signaling is critical for normal motivated behavior and reward processing (Wise and Raptis, 1986; Tsai et al., 2009; Witten et al., 2011). The release of dopamine in the NAc occurs during reward expectation and delivery for a range of stimuli (Schultz et al., 1997). Consistent with the notion that social stimuli are rewarding, the introduction of novel partners increases the release of dopamine in the NAc of rats (Robinson et al., 2002, 2011). Moreover, activity within dopamine neurons in the VTA is increased during interaction with novel, but not familiar, social partners (Gunaydin et al., 2014; Dai et al., 2022), while optogenetically increasing dopamine release within the NAc promotes investigation of familiar partners (Dai et al., 2022). Activation of dopamine D1 receptors prevents pair-bond formation in voles, while activation of dopamine D2 receptors in the NAc facilitates pair-bond formation (Aragona et al., 2005). In mice, activation of D1 receptors in the NAc facilitates social dominance in mice, whereas activation of D2 receptors does not (Van Der Kooij et al., 2018). Amphetamine, a dopamine reuptake inhibitor, and apomorphine, a dopamine D2 receptor-preferring agonist, injected into the NAc, increase social play in rats (Manduca et al., 2016). Together, these studies demonstrate that dopamine in the NAc is associated with a range of social behaviors.

Despite the robust evidence that the NAc is a critical component of the network controlling social behavior in rodent models, no studies have addressed the impact of direct manipulation of the NAc in nonhuman primates. To address this gap, we monitored dyadic social interaction in pairs of macaques following intra-NAc infusion of the GABA_A_ receptor agonist, muscimol, or the D2 receptor family agonist, quinpirole. We focused on D2 receptors due to the prosocial effects of D2 agonists observed in other species (see above). We hypothesized that both muscimol and quinpirole infusion into the NAc would increase dyadic social interactions. However, we found that inactivation of the NAc with muscimol increased social contact but this effect was only observed when the submissive, but not dominant, partner was infused. In contrast, and differing from reports in other species, modulation of D2 receptors was without effect on social behavior as measured by dyadic social interaction.

## Materials and Methods

### Experimental design

The experiments were conducted in dyads of macaques that were highly familiar with each other. The animals were implanted with a chronic drug microinfusion platform (described in detail below), and coordinates for the NAc were calculated using structural magnetic resonance imaging (MRI). We infused—on a within-subject basis—the following drugs into the NAc: muscimol (9 nmol, GABA_A_ agonist), quinpirole (70 nmol, D2/D3/D4 agonist), and saline (SAL). Animals were separated with visual access to their partners overnight prior to experimentation. Following infusion, animals were immediately transported to a social observation chamber in a room separate from the animals' home cage. Social behavior was recorded for 1 h and behaviors in Table 1 were scored in the scoring software BehaviorCloud.

**Table 1.** Operational definitions of general (nonsocial) and social behaviors observed in these experiments

Behavior	Description
General (nonsocial)	
Locomotion	Walks, runs, climbs, or jumps
Manipulation	Handles, chews, licks, moves, or smells objects or cage parts
Passive	Inactive, stays in one location
Self-directed	Engages in self-directed behaviors, i.e., self-grooms, hugs head, self-grabs and bites, presses face with hands, self-holds, closes fists, self-clutches, sexually self-stimulates, prone, or head on chest
Social	
Approach	Initiates social contact; moves body or head toward the conspecific
Isolation	Sits alone
Social contact	Any physical contact between the subject and conspecific.
Grooming-related behaviors conspecific	Subject grooms the conspecific, subject presents for grooming, subject is groomed by the conspecific

Note that our analysis ethogram also included aggression, mounting, play, withdrawal, motor stereotypies, and vocalizations, but these were either completely absent or observed at very low rates so were not formally analyzed further.

### Subjects

Nine male macaques, five rhesus macaques (*Macaca mulatta*; ME, PP/PP^, FR, MK, FI), and four pigtail macaques (*Macaca nemestrina*; JO, BA, HX, CB) were used for this experiment. These animals formed experimental pairs (Table 2). All animals, except for HX and CB, were implanted with a chronic infusion platform, which allowed a removable infusion cannula to be inserted into the target area for each infusion. For each experiment, one animal was infused with a drug (or saline) and was paired with a noninfused partner. Some animals served as each other's partners; that is, one animal was injected for some experiments and served as a noninjected partner for others (PP and ME, FR and MK), and three animals were tested only as injected subjects (FI, JO, and BA). Animals HX and CB served only as noninjected partners. Animal PP was used for two sets of experiments (labeled as cases PP and PP^). This animal was reimplanted ∼6 months after the first implant was removed. Although the partner for this animal remained the same, their baseline behaviors slightly changed, and for the new implant, a different infusion site was used. Therefore, we analyzed data from this animal as two separate cases. The number of infusions received by each subject is shown in Table 2.

**Table 2.** Table showing dyads, dominant subjects in each dyad, and the number of infusions per subject in a dyad

Infused subject	ME ○	PP □	PP^ Δ	FR ∇	MK ◊	FI ⎔	JO ⊗	BA ⊠
Partner	PP	ME	ME^	MK	FR	FR	HX	CB
Dominant	ME	ME	ME^	FR	FR	FR	JO	BA
SAL	4	2	3	3	3	3	2	4
MUS	4	2	2	4	2	2	3	3
QUIN	4	3	3	3	2	1	2	3

Animals were procured from Alpha Genesis (ME, PP), Mannheimer Foundation (FR, MK, FI), and Washington National Primate Research Center (JO, BA, HX, CB). Animals ranged in age from 4 to 6 years old and weighed between 5 and 12 kg at the time of testing. Subjects were housed in a temperature- and humidity-controlled room with a regulated 12 h light/dark cycle and maintained on a primate laboratory diet (Purina Mills, #5049) supplemented with fresh fruit, vegetables, and environmental enrichment. Water was available *ad libitum* in home cage. Animals were weighed regularly.

Animals were housed in pairs (ME and PP, JO and HX) or triads (FR, FI, and MK; BA, CB, and another animal not used here) in four connected cages, each 61 × 74 × 76 cm in size. Each pair was highly familiar at the time of testing. In parallel with this extensive socialization, animals were chair trained for drug microinfusions and box trained for transport into the social testing chamber (85 × 95 × 100 cm).

Care and housing of animals met or exceeded the standards set by the *Guide for the Care and Use of Laboratory Animals* (the Guide, National Research Council (U.S.), Institute for Laboratory Animal Research, 2011), Institute for Laboratory Animal Research recommendations, and AAALAC International accreditation standards. The study was conducted under a protocol (#2016-1115) approved by the Institutional Animal Care and Use Committee at Georgetown University.

In addition to the experiments described here, ME, PP, JO, FR, FI, MK, and BA received injections into the basolateral amygdala (for a different set of experiments), and PP, FR, and MK received injections into the bed nucleus of the stria terminalis (BNST), for a separate set of social behavior experiments (Jacobs et al., 2023).

### Implantation of drug infusion platform and site verification

Animals were implanted with a stereotaxically positioned chronic infusion platform, which enabled us to target specific sites within the NAc based on the coordinates assessed by structural MRI scans. For the postoperative MRI and the surgery, we followed procedures as described in detail in our previous studies (Forcelli et al., 2016, 2017; Wellman et al., 2016; Aguilar et al., 2018). Briefly, the infusion platform was implanted under anesthesia and aseptic conditions, followed by a postoperative regimen of analgesics and antibiotics determined in consultation with the facility veterinarian. Postoperatively, each monkey received at least one T1-weighted structural MRI scan (0.75 × 0.75 mm in-plane resolution, 1 mm slice thickness) intended to obtain coordinates for infusions in the NAc. Tungsten microelectrodes (FHC), which were visible on the scan, were used to determine the precise coordinates as described previously (Holmes et al., 2012).

### Intracerebral drug infusions

**Figure 1.**
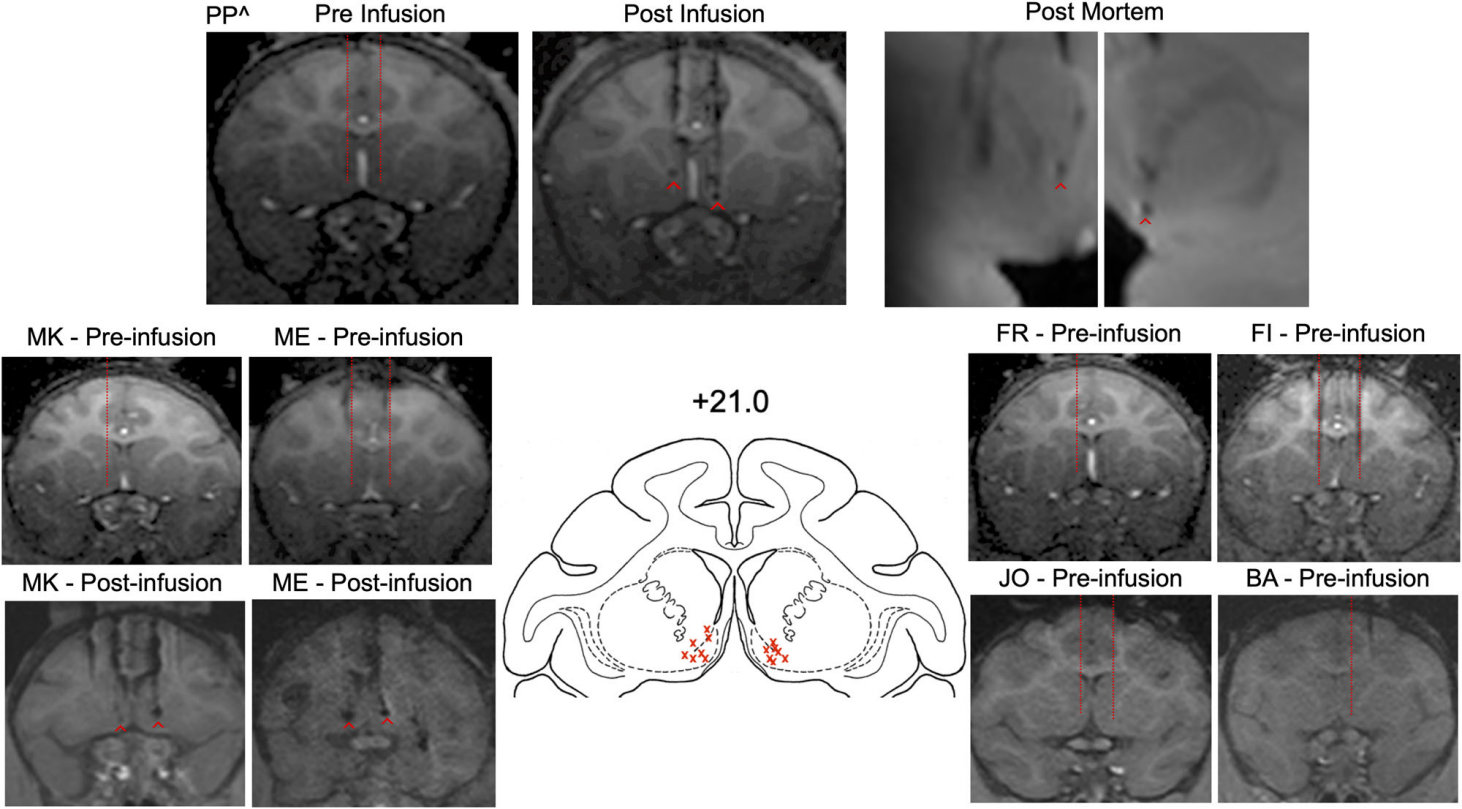
Structural MRIs showing targeting within the NAc for each infused subject. Preinfusion scans are shown for all subjects. Postinfusion scans were conducted for MK, ME, and PP^. In addition, a postmortem 7T scan was conducted on PP^. Red dotted lines show estimated coordinates for each subject on the preinfusion scan. Red ^ indicate cannula tips in the post-infusion and postmortem scans. There was signal dropoff in the left hemisphere of PP^ postmortem scan due to brain placement in the coil. The two lateral cannula tracks in the left hemisphere are the dorsal aspect of infusions aimed for the temporal cortex and amygdala. In most subjects, minimal damage occurred. Atlas plane (+21 mm from interaural plane) from the NIH Laboratory of Neuropsychology “Red” Macaque atlas with red X marks indicated plotted/estimated infusion tips from MRIs.

To transiently manipulate the NAc (for infusion sites, see Fig. 1), 9 nmol of muscimol or 70 nmol quinpirole, each in a volume of 1 µl, was infused bilaterally at a rate of 0.2 µl/min under aseptic conditions as previously described (Forcelli et al., 2017). We previously reported that this dose of muscimol produces robust, site-specific behavioral changes when infused intracerebrally in primates. These include the following: contralateral turning following unilateral infusion into the SNpr, disrupted prepulse inhibition of the acoustic startle following bilateral infusion into the deep and intermediate layers of the superior colliculus or NAc, increased social interaction following infusion into the amygdala, and increased PPI following infusion into the amygdala or substantia nigra pars reticulata (Wellman et al., 2016; Aguilar et al., 2018; Elorette et al., 2020). The dose of quinpirole was selected based on prior studies in rodents as well as a parallel study in our lab, which investigated the effect of infusion of three doses of quinpirole into the NAc on prepulse inhibition in primates (Waguespack et al., 2023). In this study, we found that the highest dose of quinpirole (70 nmol) produced the most robust effect.

This volume of drug (1 µl) was selected based on our prior infusions of the MRI contrast agent gadolinium (5 mM in saline), which resulted in an area hypersignal of ∼3 mm in diameter an hour after infusion (DesJardin et al., 2013). This volume is sufficient to cover the majority of the NAc in the primate.

The entire infusion procedure lasted 15 min, after which animals were transferred to the social testing chamber for observation. We began social observation ∼5–10 min after the microinfusion was completed. Social observation lasted 30 min. At least 48 h elapsed between drug treatments in an individual subject. Control infusions consisted of microinjection of an equivalent volume of sterile saline. Animals received between 2–4 saline infusions, 2–4 muscimol infusions, and 1–4 quinpirole infusions (Table 2).

### Analysis of social behavior

Behaviors were analyzed using the software program BehaviorCloud by two independent observers (H.W. and J.P.). General (nonsocial) and social behaviors of the infused subject were recorded immediately following drug or control infusion in a room separate from the animals' home cage. A list of analyzed behaviors is shown in Table 1.

Behaviors were classified as general (locomotion, manipulation, passive, self-directed) or social (approach, isolation, contact, grooming-related). In our prior studies, we also assessed other behaviors including motor stereotypies, vocalization, mounting, aggression, play, and withdrawal. In the present study, these were observed at very low rates (absent in most sessions) precluding formal analysis of these behaviors. Similarly, we have previously distinguished between different types of grooming-related behaviors (solicit grooming, receive grooming, groom conspecific). In the present study, solication of grooming was rarely observed, and the expression of grooming-related behaviors was variable across dyads. For this reason, we collapsed across these behaviors for statistical analysis.

One observer (H.F.W.) analyzed all videos. J.P. analyzed two complete datasets from two subjects to ensure a high level of interobserver correlation (*r* = 0.9 or better for social behaviors).

### Social dominance

To assess dominance status, we used a modified version of a previously described dominance paradigm (Mirsky et al., 1957; Malkova et al., 2010) as we have previously described (Elorette et al., 2020). In brief, each of the dyads used in this experiment was transferred to the observation chamber prior to their morning feed. Fifty food pellets (300 mg dustless precision pellets, BioServ) were dispensed at 10 s intervals into the food hopper mounted on the cage. Dominance testing was performed in the absence of any other experimental manipulations. Dominance status for each infused animal is shown in Table 2. The dominant partner was defined as the partner who retrieved the greater number of pellets.

### Data analysis and statistics

Data were analyzed using a mixed effect analysis in SPSS (version 29 with treatment and dominance status as fixed effects, session and treatment as repeated effects, and monkey as a random effect). Pairwise comparisons were corrected for multiple comparisons using the method of Sidak. GraphPad Prism 9 was used for figure preparation. Data for grooming, self-directed behavior, locomotion, social contact, and manipulation were log transformed to address values of zero and produce a model with a better fit. Statistical significance was defined as *p* < 0.05. Statistics are reported in the text, figure legends, and statistical table (Table 3).

**Table 3.** Statistical table

Manuscript reference	Figure	Data structure	Type of test	Multiple comparisons correction	Difference in estimated marginal means and 95% confidence intervals
a	Fig. 2*A*,*C*	Log-normal	Mixed effects model	Sidak	SAL v. MUS: **0.375 [0.004, 0.746]** SAL v. QUIN: −0.0630 [−0.414, 0.353]
b	Fig. 2*B*,*D*	Log-normal	Mixed effects model	Sidak	SAL v. MUS: −0.162 [−0.486, 0.161] SAL v. QUIN: −0.006 [0.746, 0.353]
c	Fig. 3*A*,*C*	Log-normal	Mixed effects model	Sidak	SAL v. MUS: **0.784 [0.066, 1.502]** SAL v. QUIN: 0.392 [−0.342, 1.126]
d	Fig. 3*B*,*D*	Log-normal	Mixed effects model	Sidak	SAL v. MUS: 0.010 [−0.617, 0.638] SAL v. QUIN: −0.076 [−0.710, 0.558]
e	Fig. 4*A*,*C*	Log-normal	Mixed effects model	Sidak	SAL v. MUS: 0.246 [−0.207, 0.700] SAL v. QUIN: **0.562 [0.081, 1.044]**
f	Fig. 4*B*,*D*	Log-normal	Mixed effects model	Sidak	SAL v. MUS: −0.100 [−0.490, 0.291] SAL v. QUIN: −0.039 [−0.440, 0.362]
g	Fig. 5*A*,*C*	Log-normal	Mixed effects model	Sidak	SAL v. MUS: −**0.444 [**−**0.858,** −**0.031]** SAL v. QUIN: 0.049 [−0.362, 0.461]
h	Fig. 5*B*,*D*	Log-normal	Mixed effects model	Sidak	SAL v. MUS: 0.077 [−0.280, 0.434] SAL v. QUIN: 0.168 [−0.189, 0.526]
i	Fig. 6*A*,*C*	Log-normal	Mixed effects model	Sidak	SAL v. MUS: −0.076 [−0.168, 0.835] SAL v. QUIN: 0.062 [−0.093, 0.957]
j	Fig. 6*B*,*D*	Log-normal	Mixed effects model	Sidak	SAL v. MUS: −0.076 [−0.673, 0.521] SAL v. QUIN: 0.432 [−0.211, 1.075]

## Results

### Localization of infusions

Infusion site targeting and verification is shown in Figure 1. In ME, PP, and MK, the calculated coordinates from the in vivo scans closely correspond to the localization of cannula tracks from the postinfusion in vivo MRI scans. No postmortem histology was available for this experiment due to the continued use of ME, FR, MK, FI, JO, and BA for behavioral experiments. We were able to obtain a postmortem MRI scan for PP, but we were not able to process the brain histologically. However, we have previously found a close correspondence between our MRI-based assessment of infusion sites and histology (Holmes et al., 2012; Forcelli et al., 2014; Wellman et al., 2016).

### Social contact

**Figure 2.**
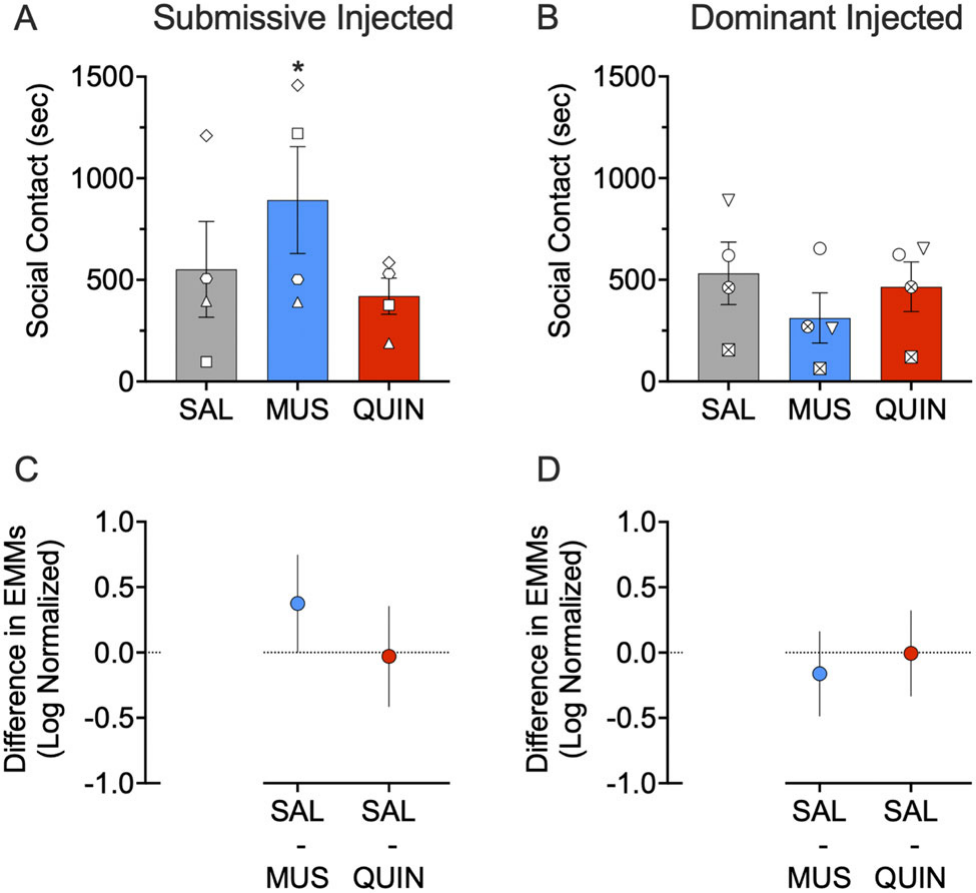
Effect of muscimol (MUS) and quinpirole (QUIN) infusion into the NAc on social contact in dominant and submissive animals. ***A***, Muscimol increases social contact in submissive animals (*p* = 0.047). ***B***, Neither muscimol nor quinpirole alter social contact in dominant subjects (*p* = 0.444 and *p* = 0.999, respectively). ***C***, Differences (and 95% confidence intervals) in estimated marginal means (log transformed) for the two treatment groups within the submissive injected subjects. ***D***, Differences (and 95% confidence intervals) in estimated marginal means (log transformed) for the two treatment groups within the dominant injected subjects. Plots provide a graphical estimation of effect size. Bars in ***A*** and ***B*** show mean + SEM. Individual data points show the average across infusions for the infused subject in each dyad. *p < 0.05, Sidak corrected; treated versus saline.

As shown in Figure 2, muscimol, but not quinpirole, infused into the NAc of the submissive subjects (*p* = 0.047; pairwise comparison, Sidak corrected) significantly increased social contact (Fig. 2*A*).*^a^* No other comparisons were statistically significant. This was reflected in a significant dominance status by treatment interaction (*F*_(2,52.826)_ = 4.137; *p* = 0.021), but neither a main effect of treatment on social contact (*F*_(2,52.826)_ = 0.733; *p* = 0.485) nor a main effect of dominance status on social contact (*F*_(1,6.194)_ = 0.994; *p* = 0.356).

Consistent with these effects, we observed no change in social contact following treatment with either muscimol or quinpirole in the dominant subjects (*p* = 0.444 and *p* = 0.999, respectively; pairwise comparison, Sidak corrected; Fig. 2*B*).*^b^* We found no effect following treatment with quinpirole in the submissive subjects (*p* = 0.979; pairwise comparison, Sidak corrected). We observed no effect with either muscimol or quinpirole on social contact when collapsed across dominance status (*p* = 0.545 and *p* = 0.983; multiple comparison, Sidak corrected).

### Grooming

**Figure 3.**
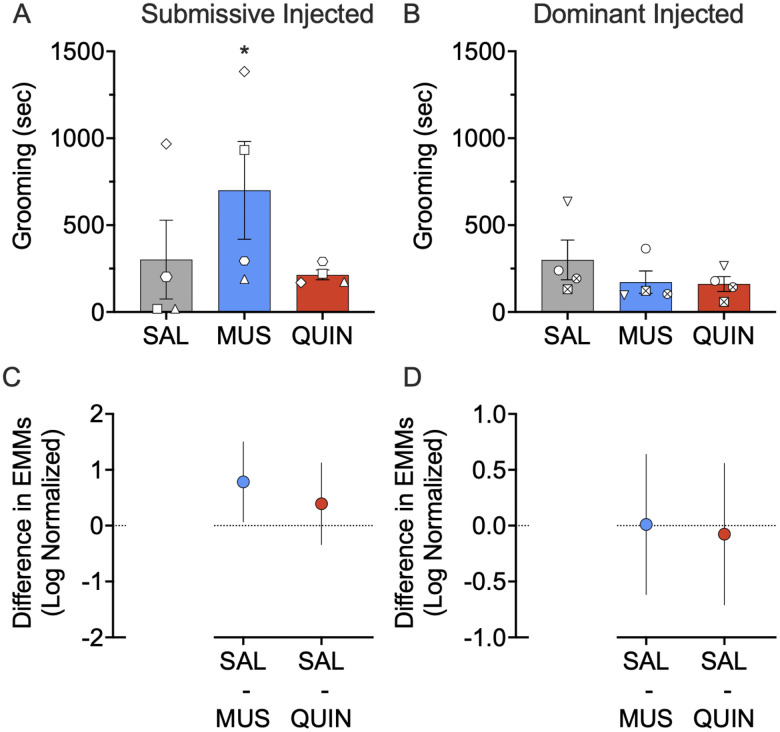
Effect of muscimol (MUS) and quinpirole (QUIN) infusion into the NAc on grooming in dominant and submissive animals. ***A***, Muscimol increases grooming-related behaviors in submissive subjects (*p* = 0.030). ***B***, Neither muscimol nor quinpirole alter grooming-related behavior in dominant subjects (*p* = 0.999 and *p* = 0.953, respectively). ***C***, Differences (and 95% confidence intervals) in estimated marginal means (log transformed) for the two treatment groups within the submissive injected subjects. ***D***, Differences (and 95% confidence intervals) in estimated marginal means (log transformed) for the two treatment groups within the dominant injected subjects. Plots provide a graphical estimation of effect size. Bars in ***A*** and ***B*** show mean + SEM. Individual data points show the average across infusions for the infused subject in each dyad. *p < 0.05, Sidak corrected; treated versus saline.

We observed a similar pattern of results when we analyzed the duration of grooming (Fig. 3). Muscimol infusion into the NAc of submissive subjects significantly increased grooming-related behaviors (*p* = 0.030; pairwise comparison, Sidak corrected; Fig. 3*A*).*^c^* No other main effects, interactions, or pairwise comparisons were statistically significant.

We observed no main effect by treatment on grooming-related behaviors (solicitation of grooming, receipt of grooming, or grooming of the conspecific; *F*_(2,49.030)_ = 1.850; *p* = 0.168), no main effect by dominance status (*F*_(1,5.671)_ = 3.099; *p* = 0.132), and no dominance status by treatment interaction (*F*_(2,49.030)_ = 1.796; *p* = 0.177). We noted no change in grooming with either infusion of muscimol or quinpirole into the dominant subjects (*p* = 0.999 and *p* = 0.953, respectively; pairwise comparison, Sidak corrected; Fig. 3*B*).*^d^* We also found no effect of quinpirole infused into the submissive subjects (*p* = 0.395; pairwise comparison, Sidak corrected; Fig. 3*A*). We found no effect of either muscimol or quinpirole on grooming-related behaviors when collapsed across dominance status (*p* = 0.117 and *p* = 0.702, respectively; pairwise comparison, Sidak corrected).

### Self-directed behavior

**Figure 4.**
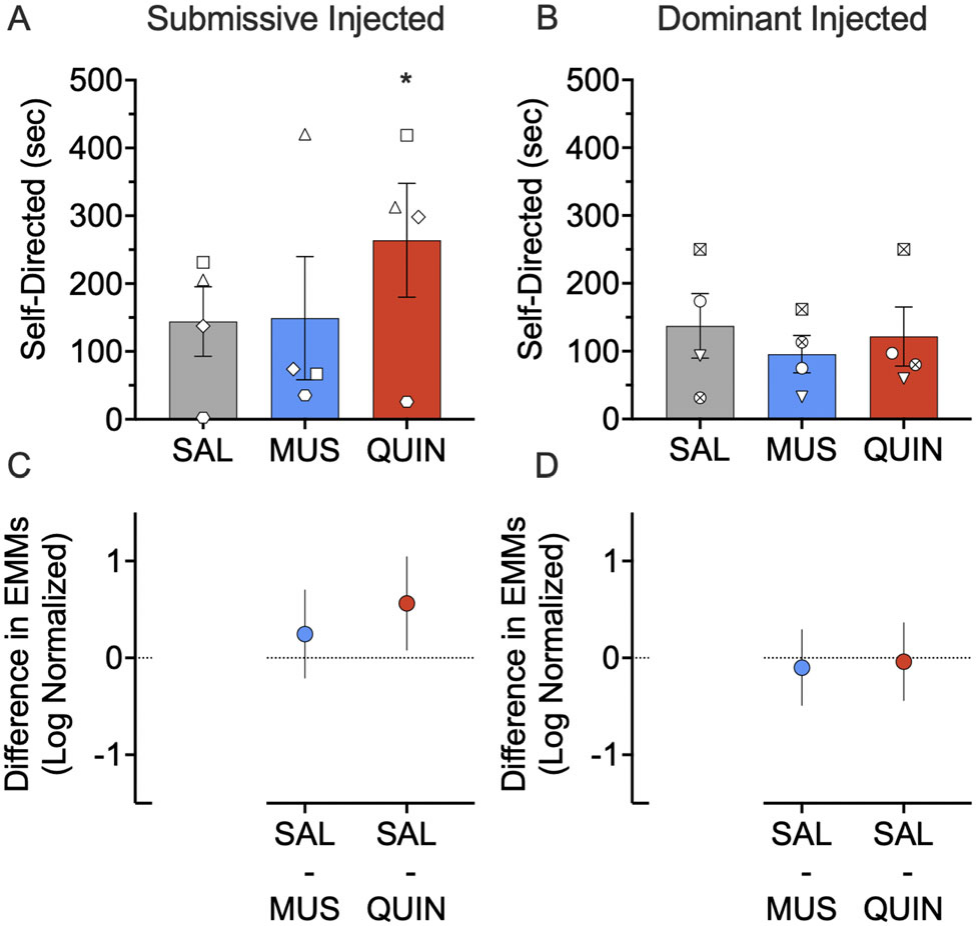
Effect of muscimol (MUS) and quinpirole (QUIN) infusion into the NAc on self-directed behavior in dominant and submissive animals. ***A***, Infusion of quinpirole into the NAc increases self-directed behavior in submissive subjects (*p* = 0.019). ***B***, Neither muscimol nor quinpirole infusion into the NAc alters self-directed behavior in dominant subjects (*p* = 0.805 and *p* = 0.969, respectively). ***C***, Differences (and 95% confidence intervals) in estimated marginal means (log transformed) for the two treatment groups within the submissive injected subjects. ***D***, Differences (and 95% confidence intervals) in estimated marginal means (log transformed) for the two treatment groups within the dominant injected subjects. Plots provide a graphical estimation of effect size. Bars in ***A*** and ***B*** show mean + SEM. Individual data points show the average across infusions for the infused subject in each dyad. *p < 0.05, Sidak corrected; treated versus Saline.

We observed no main effect by treatment on self-directed behavior (*F*_(2,52.634)_ = 1.934; *p* = 0.155), no main effect by dominance (*F*_(1,5.700)_ = 0.015; *p* = 0.907), and a borderline dominance by treatment interaction (*F*_(2,52.634)_ = 2.513; *p* = 0.091). When we followed these analyses with pairwise comparisons within submissive animals, quinpirole, but not muscimol, increased self-directed behavior in the submissive subjects (*p* = 0.019 and *p* = 0.387, respectively; Fig. 4*A*).*^e^* We found no effect with either muscimol or quinpirole when we performed pairwise comparisons in the dominant subject (*p* = 0.805 and *p* = 0.969, respectively; multiple comparison, Sidak corrected; Fig. 4*B*).*^f^* Consistent with this, we found no effect of either muscimol or quinpirole on self-directed behavior when collapsed across dominance status (*p* = 0.820 and *p* = 0.115, respectively; multiple comparison, Sidak corrected).

### Locomotion

**Figure 5.**
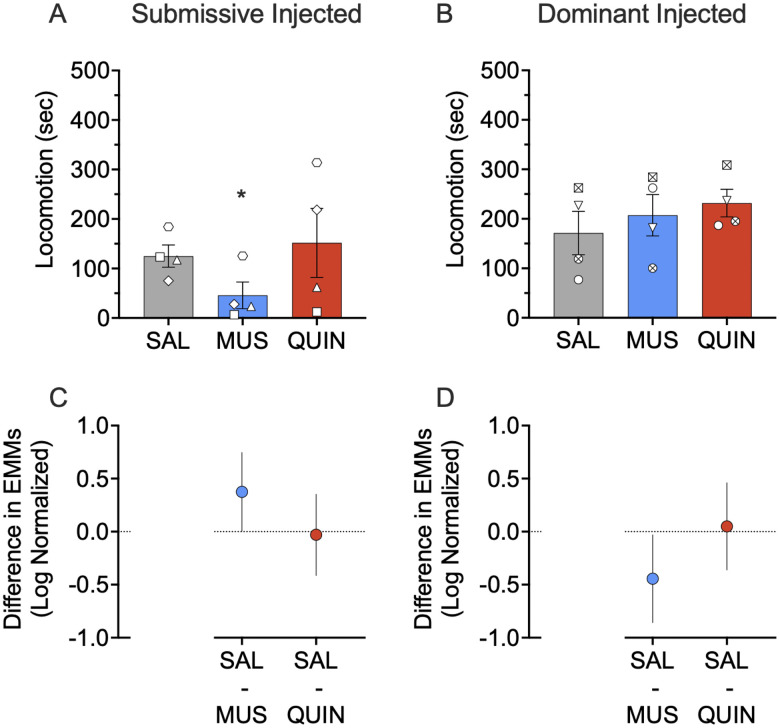
Effect of muscimol (MUS) and quinpirole (QUIN) infusion into the NAc on locomotion in dominant and submissive animals. ***A***, Muscimol infused into the NAc decreases locomotion in submissive subjects (*p* = 0.033). ***B***, Neither muscimol nor quinpirole infused into the NAc alters locomotion in dominant subjects (*p* = 0.853 and *p* = 0.480, respectively). ***C***, Differences (and 95% confidence intervals) in estimated marginal means (log transformed) for the two treatment groups within the submissive injected subjects. ***D***, Differences (and 95% confidence intervals) in estimated marginal means (log transformed) for the two treatment groups within the dominant injected subjects. Plots provide a graphical estimation of effect size. Bars in ***A*** and ***B*** show mean + SEM. Individual data points show the average across infusions for the infused subject in each dyad. *p < 0.05, Sidak corrected; treated versus saline.

As shown in Figure 5, muscimol, but not quinpirole, reduced locomotion in the submissive subject (*p* = 0.033 and *p* = 0.952; multiple comparison, Sidak corrected),*^g^* while both treatments were without effect in the dominant subject (*p* = 0.853 and *p* = 0.480, respectively; multiple comparison, Sidak corrected).*^h^* We observed a borderline effect of treatment (*F*_(2,40.712)_ = 2.828; *p* = 0.071) on locomotion, a main effect of dominance status on locomotion (*F*_(1,5.425)_ = 6.764; *p* = 0.045), and a borderline significant dominance status by treatment interaction (*F*_(2,40.712)_ = 2.584; *p* = 0.088). We observed no effect of either muscimol or quinpirole on locomotion when collapsed across dominance status (*p* = 0.236 and *p* = 0.588; multiple comparison, Sidak corrected).

### Manipulation

**Figure 6.**
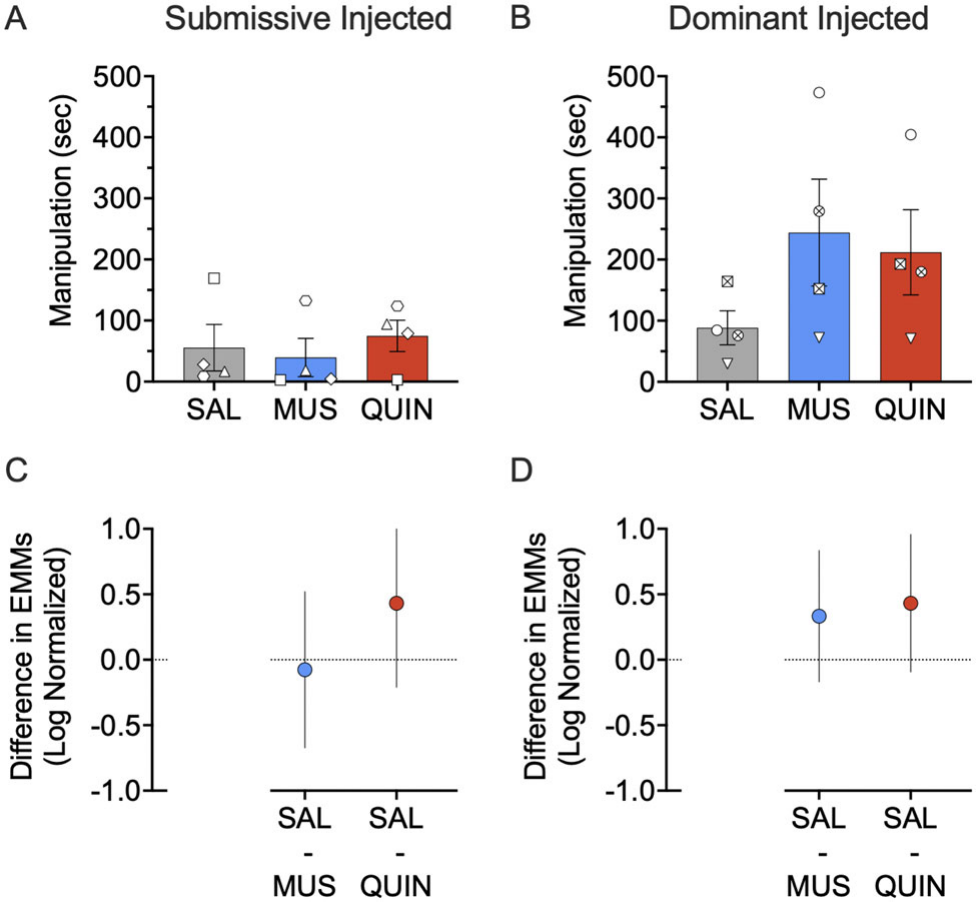
Effect of muscimol (MUS) and quinpirole (QUIN) infusion into the NAc on manipulation in dominant and submissive animals. ***A***, ***B***, Neither muscimol nor quinpirole alters manipulation in submissive or dominant subjects alone (MUS Dom, *p* = 0.245; QUIN Dom, *p* = 0.124; MUS Sub, *p* = 0.948; QUIN Sub, *p* = 0.238). ***C***, Differences (and 95% confidence intervals) in estimated marginal means (log transformed) for the two treatment groups within the submissive injected subjects. ***D***, Differences (and 95% confidence intervals) in estimated marginal means (log transformed) for the two treatment groups within the dominant injected subjects. Plots provide a graphical estimation of effect size. Bars in ***A*** and ***B*** show mean + SEM. Individual data points show the average across infusions for the infused subject in each dyad. *p < 0.05, Sidak corrected; treated versus saline.

When we collapsed across dominance status, we identified a significant increase in manipulation following quinpirole infusion (*p* = 0.039; pairwise comparison, Sidak corrected). However, as shown in Figure 6, all treatments were without effect on manipulation when assessed as a function of dominance status. We observed a borderline effect of treatment (*F*_(2,54.394)_ = 2.975; *p* = 0.059) on manipulation, a significant main effect of dominance (*F*_(1,6.455)_ = 12.170; *p* = 0.012), and no dominance by treatment interaction (*F*_(2,54.394)_ = 0.950; *p* = 0.393). We found no effect of muscimol or quinpirole in either the dominant or submissive subjects (muscimol in dominant, *p* = 0.245; quinpirole in dominant, *p* = 0.124; muscimol in submissive, *p* = 0.948; quinpirole in submissive, *p* = 0.238; pairwise comparison, Sidak corrected; (Fig. 6*A*,*B*).*^i, j^*

### Summary

Acute pharmacological inhibition of the NAc by muscimol resulted in significant behavioral differences in submissive animals but not in dominant animals. In submissive animals, we observed increase in grooming behaviors as well as in social contact, concurrent with a decrease in locomotion. None of these differences were found in dominant animals injected with muscimol. There was a slight but not significant increase in manipulation in both groups. This increase became significant only when the groups were collapsed across dominance status. A significant effect of quinpirole was also observed only in submissive animals. Quinpirole resulted in an increase in self-directed behaviors. No effects were observed in dominant animals or when the groups were collapsed across dominance status.

## Discussion

Here, we found that inactivation of the NAc with muscimol increased social contact and grooming in submissive, but not dominant, macaques. In contrast, and differing from reports in other species, the D2 receptor agonist quinpirole, when infused into the NAc, was without effect on social behavior. While decades of studies have identified a central role for the NAc in reward processing, surprisingly little is known about its role in social behavior, and our data are the first to address this in nonhuman primates.

The social repertoire of macaques is highly complex and dependent on many variables. One of these variables is dominance status. In dyads, as well as in larger groups, one animal typically displays dominant behaviors, including preferential food taking, preferred position within an enclosure, open-mouthed threats, and stares at conspecifics (Vessey, 1984). In some early studies of amygdala lesions in macaques, the impact of the lesion depended on the dominance status of the lesioned animal, and the dominance status of remaining, unlesioned, animals within the troupe (Rosvold et al., 1954). Moreover, we have found that both transient activation and transient inactivation of the amygdala reduced competitive reward seeking in dominant, but not submissive, animals (Elorette et al., 2020). These data underscore the importance of assessing the impact of brain manipulations on social behavior while taking dominance status into account.

This is particularly true for the NAc, where prior studies in a range of species have linked NAc activity to features of social dominance. Neuroimaging studies in humans have revealed increased activity in NAc when participants viewed subjects of a perceived higher rank, when participants “won” a competition with other participants, and when engaged in competition over collaboration (Bault et al., 2011; Ly et al., 2011; Kätsyri et al., 2013; Le Bouc and Pessiglione, 2013). In animal models, lesions of the NAc decreased social dominance in dominant mice (Shan et al., 2022). Similarly, transient pharmacological inactivation of the NAc decreased submissive behavior following social defeat, that is, “conditioned defeat,” in Syrian hamsters (Luckett et al., 2012). We expected that both muscimol and quinpirole infusion into the NAc in our study would modulate social behavior, in a prosocial manner. Consistent with our prior studies, we observed almost no aggression between conspecifics in a dyad; this is likely because the animals in our study are highly familiar with each other, have well-established hierarchies, and that we selected socially compatible partners for the studies. We did not measure the impact of either muscimol or quinpirole on dominance using the food competition task. Rather, drugs were only infused prior to interaction sessions without food competition. Thus, while we cannot address whether our manipulations modified dominance, per se, we observed a preferential impact of our manipulations as a function of dominance status. We observed significant effects only when the submissive subject was injected, with MUS infusion into the NAc of submissive animals increasing social contact and grooming.

The significant increases in grooming behaviors and social contact we observed are consistent with a previous study of dyadic interactions in macaques from our laboratories (Wellman et al., 2016), which showed that acute pharmacological inhibition of the basolateral amygdala by muscimol resulted in a robust significant increase in solicitation of grooming, active grooming, and social contact. A significant, but less robust, increase in these behaviors was also observed after muscimol in the central nucleus of the amygdala. More recently, we found that inhibition of the BNST resulted in an increase in social interactions, consisting mainly of passive social contact, that is, huddling, with a wider range of observed social behaviors, including grooming in some dyads (Jacobs et al., 2023). Together with the previous findings, our present results confirm the contribution of other structures connected with the amygdala, each of which may contribute to different components of the social behavior repertoire. While in the present study, we examined only male animals, social behavior in macaques differs by sex (Brown and Dixson, 2000; Hassett et al., 2010). In our prior studies of social behavior circuitry, we have not observed an interaction between sex and focal pharmacological manipulation (e.g., in the amygdala; Wellman et al., 2016).

A secondary effect of the increase in social contact and grooming in the present study was a decrease in locomotion in submissive animals. This is unsurprising given that these actions are mutually exclusive (i.e., an animal engaging in social behavior cannot be simultaneously locomoting). This finding is consistent with our previous results showing that a social interaction increase coincided with a significant decrease in locomotion after inactivation of the BNST (Jacobs et al., 2023). This decrease is likely not due to alterations to the basal ganglia motor circuit, as we did not observe a statistically significant change in locomotion when collapsed across dominance status or in dominant subjects alone (further suggesting this was due to a change in social behavior observed in submissive animals) nor did we observe any changes to locomotion following dopamine agonist (quinpirole) injection. We also report that quinpirole facilitates self-directed behavior in submissive monkeys. We noted no change in any of our analyzed behaviors following infusion of either muscimol or quinpirole into the NAc in dominant subjects. We did observe a statistically significant increase in manipulation following infusion of quinpirole into the NAc when collapsed across dominance status.

To our surprise, quinpirole infusion was without effect on social behavior when infused in either the submissive or dominant partner. We have previously reported that intra-NAc infusion of quinpirole, at the same dose and volume used in the present study, impairs prepulse inhibition of the acoustic startle response in macaques (Waguespack et al., 2023). Moreover, while quinpirole did not modulate social behavior in the present study, it did increase self-directed behaviors in submissive subjects (*p* = 0.019), as well as manipulation of cage objects when collapsed across dominance status (*p* = 0.039). This suggests that the lack of effect on social behavior is not due to insufficient drug concentration or spread.

In rodents, infusion of amphetamine (dopamine release/reuptake inhibitor) or apomorphine (dopamine receptor agonist) increases social play, whereas D1/D2 blockade reduces social play, suggesting this behavior is highly sensitive to alterations in dopamine levels at the level of the NAc in rodents (Manduca et al., 2016). Because the animals in our study were postadolescent, play is a behavior rarely observed. Whether similar infusions in juvenile macaques would modulate play remains to be explored. The lack of interaction between dominance status and quinpirole injection was also surprising and interesting in light of evidence suggesting that dominant macaques, when socially housed, have higher D2 receptor expression than do submissive animals (Morgan et al., 2002).

A range of papers have demonstrated increased dopamine transients in the NAc (Robinson et al., 2002, 2011) or increased activity of VTA to NAc-projecting dopamine neurons (Gunaydin et al., 2014) during interactions with novel conspecifics. In our study, animals were highly familiar and lived in an established social hierarchy, which may have decreased the impact of dopamine on their social interactions. Given the propensity for aggression between unfamiliar macaques, testing the impact of social novelty would not be prudent.

Despite the central role that the NAc plays in reward processing, and despite mounting evidence from rodent studies, the role of the NAc in social behavior in the nonhuman primate brain has been essentially unstudied. Given the many differences we and others have observed with respect to the impact of brain manipulations on behavior between rodents and primates (DesJardin et al., 2013; Dybdal et al., 2013; Aguilar et al., 2018; Waguespack et al., 2023), cross-species comparisons are both warranted and sometimes surprising. While our findings with muscimol infusion were in the present study were generally consistent with the effects reported in rodents, much to our surprise, quinpirole infusion was entirely without effect on social behavior. Together, these data provide the first evidence for a contribution of the NAc to the neural circuitry controlling social behavior in nonhuman primates and demonstrate that suppression of activity within the NAc increases affiliative interactions between familiar conspecifics.

## Synthesis

Reviewing Editor: Sam Golden, The University of Washington Decisions are customarily a result of the Reviewing Editor and the peer reviewers coming together and discussing their recommendations until a consensus is reached. When revisions are invited, a fact-based synthesis statement explaining their decision and outlining what is needed to prepare a revision will be listed below. The following reviewer(s) agreed to reveal their identity: NONE. Note: If this manuscript was transferred from JNeurosci and a decision was made to accept the manuscript without peer review, a brief statement to this effect will instead be what is listed below.

Dear Dr. Malkova and Forcelli,

Thank you for the transferred manuscript from The Journal of Neuroscience. I have reviewed the prior reviewers comments, your rebuttal, and the revised manuscript. Your revised manuscript is appropriate for publication without new reviews, which I believe would likely be repetitive and not entirely helpful. I look forward to seeing your manuscript in print.
